# La réponse humorale contre les peptides antigéniques de *Plasmodium falciparum* (MSP1, MSP2 et SR-11.1) chez des sujets vivant en zone endémique

**DOI:** 10.11604/pamj.2022.41.250.30950

**Published:** 2022-03-28

**Authors:** Yacouba Sourabié, Yétéma Dieudonné Yonli, Francis Fumoux, Yves Traoré

**Affiliations:** 1 Département des Sciences Fondamentales et Mixtes, Université Nazi Boni, Bobo Dioulasso, Burkina Faso,; 2 Centre National de Transfusion Sanguine (CNTS), Ouagadougou, Burkina Faso,; 3 Faculté des Sciences de Luminy, Aix Marseille Université, Marseille, France,; 4 Unité de Formation et de Recherche Science de la Vie et de la Terre, Université Joseph Ki-Zerbo, Centre de Recherche en Sciences Biologiques Alimentaires et Nutritionnelles (CRSBAN), Ouagadougou, Burkina Faso

**Keywords:** IgG, MSP1, MSP2, SR-11.1, *Plasmodium falciparum*, IgG, MSP1, MSP2, SR-11.1, Plasmodium falciparum

## Abstract

**Introduction:**

les vaccins constituent les outils privilégiés pour le contrôle et l´éradication du paludisme dans le monde. Actuellement, la recherche et développement est orientée vers trois types de candidats vaccins qui ciblent différents stades de vie du Plasmodium falciparum chez l´homme et le vecteur. L´objectif est d´évaluer la réponse humorale contre les peptides antigéniques de Plasmodium falciparum (MSP1, MSP2 et SR-11.1) chez des sujets vivant en zone endémique.

**Méthodes:**

nous avons réalisé une étude transversale sur une période de 5 mois portant sur les sérums de 182 sujets Vietnamien vivant dans les aires endémiques. Le sang total a été centrifugé et, le sérum aliquoté dans des cryotubes et conservé à -20°C pour le dosage des immunoglobulines G (IgG). Le dosage des immunoglobulines G totales a été réalisé par la technique ELISA après couplage des peptides avec le glutaraldéhyde.

**Résultats:**

au total, 182 sérums de sujets Vietnamiens vivant en zone endémique ont été inclus. Dans les différentes tranches d´âges d´études, les IgG totales spécifiques contre les peptides antigéniques (MSP-1; MSP-2 et SR-11.1) de P. falciparum montrent une répartition âge dépendant. Chez les sujets de 3 à 19 ans, le taux des IgG totales spécifiques contre Plasmodium falciparum est moins élevé par rapport à celui des groupes d´âge de plus de 20 ans (p=0.07). La comparaison des taux d´IgG spécifiques contre les peptides antigéniques MSP-1, MSP-2 et SR-11.1 du P. falciparum dans les groupes d´âge, donne une moyenne significativement plus élevée pour MSP-1 et MSP-2 par rapport au taux d´anticorps anti-SR-11.1 (p=0.04).

**Conclusion:**

nous avons trouvé que le taux des anticorps spécifiques contre les peptides antigéniques (MSP-1; MSP-2 et SR-11.1) de P. falciparum est âge dépendant. Des trois peptides antigéniques, MSP-1 et MSP-2 apparaissent plus immunogènes que SR.11.1. Donc, SR.11.1 est une macromolécule pour le système immunitaire et de ce point de vue apparait nettement moins immunogène que les autres peptides.

## Abstract (en)

**Introduction:**

vaccines are the key tools for controlling and eradicating malaria worldwide. Currently, research and development are focused on three types of vaccine candidates that target different life stages of Plasmodium falciparum in humans and the vector. The purpose of this study is to evaluate the humoral response to Plasmodium falciparum antigenic peptides (MSP1, MSP2 and SR-11.1) in subjects living in endemic areas.

**Methods:**

we conducted a cross-sectional study of serum samples collected from 182 Vietnamese subjects living in endemic areas over a period of 5 months. Whole blood was centrifuged and the serum was aliquoted into cryovials and preserved at -20°C until IgG testing. ELISA was used for total immunoglobulin G (IgG) antibodies testing after peptide coupling with glutaraldehyde.

**Results:**

a total of 182 sera samples from Vietnamese subjects living in endemic areas were included. In the different study age groups, total antigen-specific IgG antibodies against Plasmodium falciparum antigenic peptides (MSP-1; MSP-2 and SR-11.1) showed age-dependent distribution. In subjects aged 3-19 years, the level of total antigen-specific IgG antibodies against Plasmodium falciparum were lower than those of the age groups over 20 years of age (p=0.07). Comparison of specific antigen-specific IgG antibody levels with Plasmodium falciparum antigenic peptides MSP-1, MSP-2, and SR-11.1 in the age groups showed a significantly higher mean in MSP-1 and MSP-2 compared to anti-SR-11.1 (p=0.04).

**Conclusion:**

this study highlights that the level of specific antibodies against Plasmodium falciparum antigenic peptides (MSP-1; MSP-2 and SR-11.1) is age dependent. Of the three antigenic peptides, MSP-1 and MSP-2 appear to be more immunogenic than SR.11.1. Therefore, SR.11.1 is a macromolecule to the immune system and, from this point of view, appears to be significantly less immunogenic than other peptides.

## Introduction

Le paludisme est un véritable problème de santé publique. Des progrès notables ont été effectués dans plusieurs domaines de lutte contre *Plasmodium falciparum* sans pouvoir diminuer le nombre de cas de paludisme dans le monde [1]. D´où la nécessité de nouvelles approches pour le contrôle et l´éradication de la maladie. Les vaccins constituent à cet effet les outils privilégiés de lutte contre ce fléau. Dès lors, les travaux se sont focalisés sur les antigènes plasmodiaux de trois stades: les peptides antigéniques du stade pré-érythrocytaires, les peptides antigéniques du stade érythrocytaires et les peptides antigéniques des stades sexués chez l´homme et chez le vecteur [2,3]. Plusieurs études immuno-épidémiologiques ont montré que la protection contre le paludisme est médiée au moins partiellement par les anticorps [4,5]. La fécondation du parasite peut être bloquée par les anticorps, lorsque le repas sanguin du moustique contient des anticorps dirigés contre les formes sexuées du parasite, en même temps que des gamétocytes. Les sous classes d´IgG agissent sur la gamétocytogénèse en induisant l'agglutination et la lyse des gamétocytes par le complément [6,7] et en inhibant la transmission des gamétocytes aux anophèles [8,9].

Ainsi, la megaproteïne SR-11.1 est considéré comme un potentiel candidat vaccin parce qu´il induit la production d´anticorps qui peuvent neutraliser les sporozoïtes de *Plasmodium falciparum* [10]. Egalement, MSP1 et MSP2 sont considérés comme de potentiels candidats vaccins parce qu´ils induisent la production d´anticorps qui peuvent inhiber l'invasion des érythrocytes par les mérozoïtes [11]. Les sous classes d´IgG coopèrent avec les cellules effectrices, éliminent ou inhibent la croissance des parasites [12] au stade intra-érythrocytaire [13] ou au stade intracellulaire des cellules hépatiques [14]. C´est dans ce but que nous avons évalué la réponse humorale contre les peptides antigéniques de *Plasmodium falciparum* (MSP1, MSP2 et SR-11.1) chez des sujets vivant en zone endémique au centre du Vietnam.

## Méthodes

Il s´agit d´une étude transversale sur une période de 5 mois portant sur les sérums de 182 sujets Vietnamien vivant dans les aires endémiques. Ces sujets vivent dans la commune de Xa Thanh, district de Huong Hoa. Cette commune est située dans une région d'altitude, le long de la rivière Sê Pôn qui sert de frontière entre le Laos et le Viêt-Nam. Ils sont essentiellement agriculteurs et éleveurs. C'est une zone dont le développement économique est particulièrement difficile, le revenu annuel en 2002 était de l'ordre de 35 USD/personne/an. Le niveau d'instruction demeure faible. La population pratique des cultures intermittentes chaque année en utilisant des zones de brûlis à distance de l'habitat permanent. Ils passent fréquemment la nuit dans les forêts. L'habitat est majoritairement traditionnel, l´utilisation des moustiquaires est limitée en raison des craintes d'incendie.

L´analyse au laboratoire s´est déroulée à la faculté de pharmacie: unité mixte de recherche-MD3 (relation hôte-parasites immunogénétique et pharmacothérapeutique). Nos sérums provenaient de sang total de sujets Vietnamien chez qui, il a été réalisé depuis 2002 une ponction veineuse. Le sang a été centrifugé, aliquoté dans des cryotubes et conservé à 20°C pour le dosage des IgG.

**Les anticorps:** les anticorps suivants ont été utilisés: anticorps monoclonaux de souris anti-IgG1 humain couplés à la phosphatase alcaline (Becman Coulter®, clone 4E3, réf 733176); anticorps monoclonaux de souris anti-IgG3 humain couplés à la phosphatase alcaline (Becman Coulter®, clone HP6050, réf 733221) et les anticorps polyclonaux de chèvre anti-Ig G totales humain couplés à la phosphatase alcaline (Becman Coulter®, réf 732542).

**Peptides synthétiques:** trois peptides synthétiques correspondant à des régions de forte conservation des épitopes B et reconnus par des anticorps des individus vivant dans des zones d´endémie ont été utilisés. Il s´agit des peptides: MSP-1 -(KLYQAQYDLSF) représente des acides aminés de la position 277 à 287 de la partie conservée de N-terminal de MSP-1; MSP-2-(KAASNTFINNA) représente des acides aminés de la position 27 à 34 de la partie conservée de N-terminal de MSP-2. Il est très hydrophobe. SR11.1-(EEVVVVLIEEVIPEELVL) représente l´unique subrégion de la séquence de la megaproteïne Pf11.1. C´est un antigène qui est exprimé au stade sporozoïte et au stade hépatique de *Plasmodium falciparum*. Ces antigènes plasmodiaux ont été synthétisés et fournis sous forme poudre par le laboratoire ALTERGEN® (France).

**Dosages des immunoglobulines totales par Enzyme-linked Immuno Sorbent Assay (ELISA) après couplage des peptides avec le glutaraldéhyde:** la sensibilisation directe par des peptides synthétiques sur la plaque ELISA est en générale peu efficace et dépend beaucoup de leur taille et de leur charge. Pour optimiser la sensibilité de notre test, nous avons couplé les peptides antigéniques au glutaraldéhyde en utilisant la poly-l-lysine hautement polymérisée comme résine. La poly-l-lysine est sensibilisée sur la plaque ce qui sert de point d´ancrage en exposant le groupement NH3+de ses chaines latérales aux fonctions CHO réactives du glutaraldéhyde. La liaison covalente formée après réaction chimique permet alors de fixer l´agent de couplage à la plaque. Une seconde réaction entre la deuxième fonction aldéhyde du glutaraldéhyde et une fonction amine primaire (NH3+ terminal ou latérale) permet la fixation covalente des antigènes plasmodiaux (MSP1, MSP2 et SR11.1) à la poly-l-lysine et donc une meilleure fixation à la plaque ELISA. La fonction amine de la glycine a été ensuite utilisée pour bloquer les fonctions aldéhydes.

Nous avons suivi le protocole suivant: la poly-l-lysine est sensibilisée une nuit à 4°C (40μg/ml dans du tampon carbonate pH=9.6) à raison de 100μl/puits. La plaque est lavée trois fois au PBS 1X, puis 100μl/puits de glutaraldéhyde 1% dilué dans du tampon PBS 1X sont incubés pendant 30mn à la température ambiante. La plaque est ensuite lavée trois fois et la solution de peptides antigéniques (10μg/ml dilué dans le tampon PBS 1X) est ajoutée dans les puits (100μl/puits). La plaque est incubée la nuit à la température ambiante, lavée trois fois puis les fonctions réactives libres sont bloquées par la glycine 1M (dans du PBS 1X, 200μl/puits) pendant 1h à la température ambiante. Enfin la plaquée est lavée et saturée au tampon phosphate salin (PBS)-Lait 3% (2h à la température ambiante, 250μl/puits). Puis nous avons suivi les étapes décrites dans le dosage des IgG totales après la saturation de la plaque.

**Figure 1:**
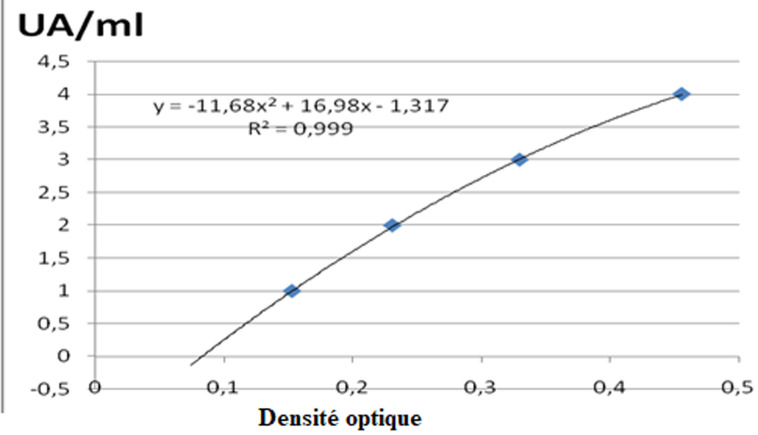
courbe de tendance obtenue avec le pool de sérums Africains (cas du MSP-1)

**Figure 2:**
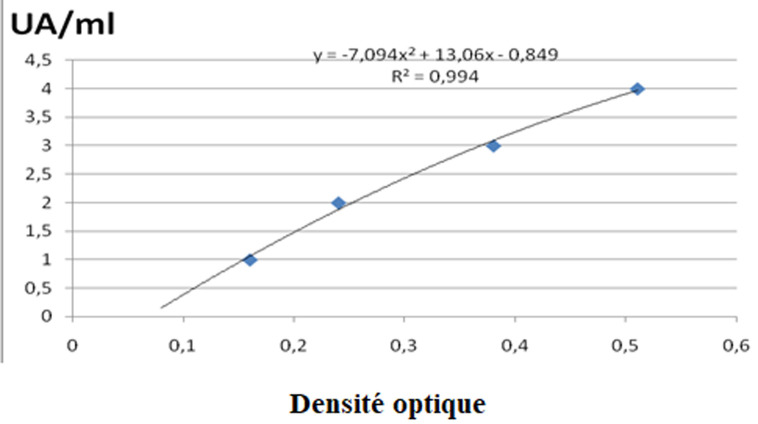
courbe de tendance obtenue avec le pool de sérums Africains (casdu MSP-2)

**Figure 3:**
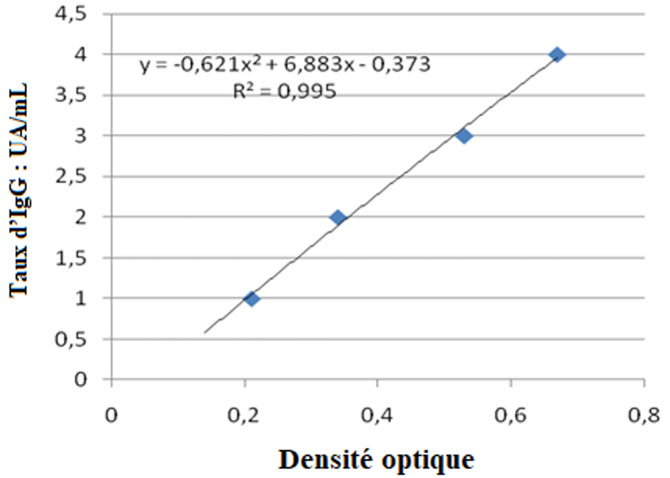
courbe de tendance obtenue avec le pool de sérums Africains (cas du SR.11.1)

**Traitement et analyse des données:** a toutes les valeurs de densité optique, il a été soustrait la densité optique du «blanc». Afin de comparer les valeurs obtenues entre les différentes manipulations, nous avons standardisé les valeurs du pool positif. Cette correction a ensuite été appliquée à la densité optique de chaque sérum. Le titrage des échantillons est réalisé à l´aide d´une gamme obtenue à partir d´un pool de sérums de sujets Africains vivant en zone endémique. Les facteurs de dilutions suivantes ont été utilisés: 20; 50; 100; 200 et 400. L´absorbance mesurée pour la dilution 1/400 a été associée à la valeur de 10 UA/ml. L´équation de la courbe de tendance obtenue à partir de la gamme nous a permis de calculer pour chaque absorbance mesurée son équivalent en UA/ml (Figure 1, Figure 2, Figure 3). Les tests utilisés pour la comparaison des moyennes étaient le test de Khi deux et Kruskal Wallis. Les valeurs de p<0.05 étaient statistiquement significatives.

## Résultats

**Caractéristiques socio-démographiques:** l´étude a été réalisée sur 182 sérums de sujets Vietnamiens, appartenant à 35 familles, dont le sang a été prélevé en 2002. Toutes ces personnes étaient asymptomatiques cliniques au paludisme au moment du prélèvement. Ces personnes vivent dans la commune de Xa Thanh. La répartition de l´âge est de 3 à 79 ans, le sex-ratio H/F est de 1.6. Nous avions reparti les individus en quatre tranches d´âge. Dans ces différents groupes d´âge, on observe une répartition similaire (Tableau 1).

**Tableau 1:** répartition des sujets selon l’âge

Tranche d'âge (ans)	Effectifs	Pourcentage (%)
3-10	47	25.82
11-19	52	28.57
0-31	43	23.62
Plus de 31	40	21.97
Total	182	100

**Taux des IgG totales en fonction du peptide antigénique et l´âge:** dans nos divers groupes d´âges d´études, les IgG totales spécifiques contre les peptides antigéniques (MSP-1; MSP-2 et SR-11.1) de *P. falciparum* montrent une répartition âge dépendant. Chez les sujets de 3 à 19 ans, le taux des IgG totales spécifiques contre *Plasmodium falciparum* est moins élevé par rapport à celui des groupes d´âge de plus de 20 ans (p=0.07) (Tableau 2, Figure 1, Figure 2, Figure 3). La comparaison des taux d´IgG spécifiques contre les peptides antigéniques MSP-1, MSP-2 et SR-11.1 du *P. falciparum* dans les groupes d´âge, donne une moyenne significativement plus élevée pour MSP-1 et MSP-2 par rapport au taux d´anticorps anti-SR-11.1 (p =0.04) (Tableau 2).

**Tableau 2:** taux des IgG en fonction du peptide antigénique et l’âge

Tranche d'âge (ans)	Taux d'IgG totales (UA/ml)		
	Anti-MSP1	Anti-MSP2	Anti-SR11.1
3-10	40.6 ± 0.152	33.3 ± 0.141	29.5 ± 0.161
11-19	41 ± 0.171	35.5 ± 0.133	33.7 ± 0.149
20-31	52.7 ± 0.177	44.8 ± 0.135	42.3 ± 0.240
32 et plus	58.5 ± 0.163	48 ± 0.43	45.3 ± 0.229

## Discussion

Les mécanismes de protection des anticorps contre le paludisme ont fait l'objet de nombreuses études. Nous avons présenté dans ce présent travail une première évaluation de la réponse humorale contre différents Ag de *P. falciparum* chez les individus vivant dans une région endémique au centre du Viet Nam. Les anticorps anti *P. falciparum* augmentent graduellement avec l'âge de 3 à 19 ans. Chez les plus de 20 ans, le taux d´Immunoglobulines G totaux contre les peptides antigéniques de *Plasmodium falciparum* (MSP1, MSP2 et SR-11.1) sont élevés. Cela peut être dû à une lente acquisition de l´immunité chez cette population vivante en zone d´endémie palustre à cause d´une diversité génétique des parasites. Le développement et l'acquisition de l'immunité protectrice contre le paludisme se déroulent en quelques années après plusieurs infections répétées au parasite. L'accélération de l'immunité dépend aussi des niveaux de transmission dans les zones endémiques [15]. De plus, l'âge a été rapporté comme un facteur de forte influence sur la charge parasitaire sanguine et sur les accès palustres [16]. En effet, l´âge est considéré comme un facteur principal influençant la production des IgM, IgG2 et IgG3 anti *P. falciparum* [17]. Ces données suggèrent que certains paramètres dépendants de l'âge sont impliqués dans le contrôle de l'infection et de la maladie.

Concernant les peptides antigéniques, nous avons remarqué que la reconnaissance des anticorps anti SR.11.1, MSP-1 et MSP-2 pour les individus de notre étude est beaucoup plus faible. Ces faibles réactivités rendent difficile l´évaluation d´une relation nette dans les groupes d´âge. Aussi nous avons trouvé que les taux moyens des IgG spécifiques contre MSP1 et MSP-2 sont significativement plus élevés par rapport au taux moyen de SR.11.1 dans les groupes d´âge.

Ce résultat pourrait s´expliqué par la variabilité des choix de réponse à de nombreux antigènes des souches parasitaires [18]. Les taux d´isotypes spécifiques de certains antigènes de *P. falciparum* ne peuvent pas être considérés comme des valeurs prédictives de la protection contre l´infection ou contre la maladie, car des études immunologiques ont montré qu´en fonction des différentes sous classes d´IgG spécifiques de certains antigènes, celles-ci peuvent être impliquées dans la protection [19,20]. Concernant la SR.11.1, c´est une macromolécule pour le système immunitaire et de cet point de vue apparait nettement moins immunogène que les autres peptides [10].

## Conclusion

Nous avons trouvé que le taux des anticorps spécifiques contre les peptides antigéniques (MSP-1; MSP-2 et SR-11.1) de *P. falciparum* est âge dépendant. Aussi, pour les trois peptides antigéniques, MSP-1 et MSP-2 apparaissent plus immunogènes que SR.11.1. Donc, SR.11.1 est une macromolécule pour le système immunitaire et de ce point de vue apparait nettement moins immunogène que les autres peptides.

### Etat des connaissances sur le sujet

- Le contrôle et l´éradication du paludisme passe par la mise au point d´un vaccin;
- La recherche est orientée vers trois types de candidats vaccins qui ciblent différents stades de vie du Plasmodium falciparum chez l´homme et le vecteur;
- Les études immuno-épidémiologiques sur les peptides antigéniques qui induisent une réponse immunitaire efficace sur plasmodium falciparum sont insuffisantes.

### Contribution de notre étude à la connaissance

- Ce travail permettra d´identifier les mécanismes immuns impliqués dans la protection contre le plasmodium et donc déterminer le meilleur candidat vaccin contre le paludisme.

## Remerciements

Nous remercions les sujets de Xa Thanh qui ont bien voulu participer à cette étude. Nous tenons à remercier les laboratoires Altergen® pour avoir synthétiser les peptides antigéniques d´intérêt, Beckam Coulter® pour la production des anticorps monoclonaux d´origine murine et de chèvre. Des remerciements particuliers au responsable de l´Unité Mixte de recherche-MD3, Aix Marseille Université, France pour avoir accepté le déroulement de l´étude.
